# Do Current Fortification and Supplementation Programs Assure Adequate Intake of Fat-Soluble Vitamins in Belgian Infants, Toddlers, Pregnant Women, and Lactating Women?

**DOI:** 10.3390/nu10020223

**Published:** 2018-02-16

**Authors:** Isabelle Moyersoen, Carl Lachat, Koenraad Cuypers, Karin De Ridder, Brecht Devleesschauwer, Jean Tafforeau, Stefanie Vandevijvere, Margot Vansteenland, Bruno De Meulenaer, John Van Camp, Herman Van Oyen

**Affiliations:** 1 Department of Public Health and Surveillance, Scientific Institute of Public Health (WIV-ISP), Juliette Wytsmanstraat 14, 1050 Brussels, Belgium; Koenraad.Cuypers@wiv-isp.be (K.C.); Karin.DeRidder@wiv-isp.be (K.D.R.); brecht.devleesschauwer@wiv-isp.be (B.D.); Jean.Tafforeau@wiv-isp.be (J.T.); stefanie.vandevijvere@wiv-isp.be (S.V.); herman.vanoyen@wiv-isp.be (H.V.O.); 2 Department of Food Safety and Food Quality, Ghent University, Coupure Links 653, 9000 Ghent, Belgium; Carl.lachat@Ugent.be (C.L.); Margot.vansteenland@UGent.be (M.V.); bruno.demeulenaer@UGent.be (B.D.M.); john.vancamp@ugent.be (J.V.C.); 3 Department of Public Health, Ghent University, De Pintelaan 185, 9000 Gent, Belgium

**Keywords:** infants, toddlers, pregnant women, lactating women, dietary intake, fat-soluble vitamins, micronutrient adequacy, excessive intake, fortified foods, supplements, Belgium

## Abstract

Adequate intakes of fat-soluble vitamins are essential to support the growth and development of the foetus, the neonate, and the young child. By means of an online self-administered frequency questionnaire, this study aimed to evaluate the intake of vitamins A, D, E, and K in Belgian infants (*n* = 455), toddlers (*n* = 265), pregnant women (*n* = 161), and lactating women (*n* = 165). The contribution of foods, fortified foods, and supplements on the total intake was quantified. 5% of toddlers, 16% of pregnant women, and 35% of lactating women had an inadequate vitamin A intake. Conversely, excessive vitamin A intakes were associated with consumption of liver (products). Furthermore, 22% of infants were at risk for inadequate vitamin D intake due to the lack of prophylaxis, while consumption of highly dosed supplements posed a risk for excessive intakes in 6%–26% of infants. Vitamin D intake in pregnant women and lactating women was inadequate (median of 51%, respectively, 60% of the adequate intake). In all groups, the risk for inadequate intake of vitamin E and K was low. Contribution of fortified foods to vitamin A, D, E, and K intake was minor, except in toddlers. National fortification strategies should be investigated as an alternative or additional strategy to prevent vitamin D and A deficiency. There is a need to revise and set uniform supplement recommendations. Finally, non-users of vitamin D prophylaxis need to be identified for targeted treatment.

## 1. Introduction

Fat-soluble vitamins (vitamin A, D, E, and K) play a vital role in growth and development, and, consequently, are crucial micronutrients in pregnant women and lactating women, the neonate, and the young child. Nutrient requirements increase substantially during pregnancy and lactation to support maternal metabolism, tissue development, and foetal and infant growth [1,2,3,4,5].

Deficiencies in fat-soluble vitamins during reproduction and lactation are associated with different adverse health outcomes in the mother and the neonate. These include intrauterine and postnatal growth retardation, congenital malformations (vitamin A deficiency), maternal osteomalacia, infant hypoplasia of tooth enamel, neonatal hypocalcaemia, tetany (vitamin D deficiency), and haemorrhage (vitamin K deficiency) [1,2,3,4,5].

Additionally, low maternal vitamin status further predisposes low infant stores at birth and low vitamin intake from human milk. Lastly, transfer of vitamin D and vitamin K through breast milk is limited and requires dietary strategies to ensure optimal supply for neonates [6,7].

Vitamin A and D deficiencies are ubiquitous in Europe [8,9,10]. However, only a few dietary intake studies describe vitamin A and D deficiencies in vulnerable population subgroups, such as young children, pregnant women, and lactating women [2,3,8,11,12,13]. National studies often omit these groups, as they are difficult to reach and require adapted dietary assessment methods [14,15]. After an extensive literature review, dietary intake data on vitamins A, D, E, and K in Belgian infants, toddlers, pregnant women, and lactating women was found to be limited. Nevertheless, Belgian dietary studies in young children (2.5–6 years) and women of reproductive age revealed inadequate vitamin A and D intake [11,16]. Inadequate consumption was related to unbalanced food consumption and unsatisfactory supplement compliance [16]. Plasma 25-hydroxyvitamin D (S-25(OH)D) is a biomarker for vitamin D status and reflects the availability of vitamin D in the body from both dietary and endogenous sources [8]. A Belgian study on vitamin D status among a representative sample of more than 1000 pregnant women revealed vitamin D deficiency (S-25(OH)D < 20 ng/mL) in 45% of the subjects, from which 12% were severely vitamin D-deficient (S-25 (OH)D < 10 ng/mL) [17].

At national level, it is important to imply strategies for increasing micronutrient intakes of population groups at risk for inadequacies. Formula milk is enriched with vitamins A, D, E, and K in accordance with national and European regulations to cover the needs of fat-soluble vitamins in non-breastfed infants aged 0–12 months [18,19]. Margarines and spreadable fats are mandatorily fortified with vitamins A and D to obtain similar levels as in butter [20]. Prophylaxis of vitamin D in the young child and mother, and vitamin K prophylaxis at birth and during lactation, are further widespread precautions to prevent vitamin D and K deficiencies. However, these levels of prophylaxis differ greatly between countries due to differing objectives, such as avoidance of deficiency diseases or prevention of suboptimal status [18,21]. Furthermore, the applied strategies might not reach all vulnerable subgroups or may still be inadequate and, consequently, require frequent monitoring to allow for possible adjustments.

Despite deficiencies, the aggregated intake from fortified foods and supplements raises concerns for excessive intakes of fat-soluble vitamins in high-consumers of such products [5]. Excessive intakes should be carefully monitored to avoid development of adverse health outcomes, such as bulging fontanel in infants, increased intracranial pressure, teratogenic effects, and hepatoxicity (vitamin A); hypercalcaemia and hypercalciuria (vitamin D); and blood clotting (vitamin E) [22].

The challenge for nutrition policymakers is to provide an entire population with adequate amounts of a nutrient while accounting for the special needs of vulnerable groups, meanwhile preventing excessive intakes in high consumers. To address this challenge, we initiated the VITADEK study—i.e., a Belgian study on the intake of vitamin A, D, E, and K [14]. This study was unique as it aimed to evaluate the adequacy of fat-soluble vitamins in four vulnerable groups: infants, toddlers, pregnant women, and lactating women. In addition, the study aimed to describe the contribution of foods, both mandatorily and voluntarily fortified, and supplements on the intake of vitamins A, D, E, and K. Intake data from breastfed and formula-fed infants were evaluated separately to account for the effect of formula-milk versus breast milk compositions. Along with the data of the general Belgian population, the study aimed to present a global picture on vitamin A, D, E, and K intake in Belgium [16]. The joint results will allow for an effectiveness evaluation of current Belgian nutritional policies and provide a baseline for formulating new nutritional recommendations. In a European context, our study is unique as it provides a broad picture of how intake distributions of fat-soluble vitamins might differ in particular subgroups of a population.

## 2. Materials and Methods

### 2.1. Data Collection

The target population of the VITADEK-study was comprised of infants (0–11 months), toddlers (12–35 months), and pregnant women and lactating women residing in Belgium.

The survey methodology is described in detail in a paper by Moyersoen et al. [14]. Briefly, the study adopted a cross-sectional design with a multi-stage sampling procedure, including a stratification according to provinces (*N* = 11) and a systematic selection of 30 municipalities stratified by province. Recruitment of infants, toddlers, and lactating women occurred in the child health consultation centre (CHCC) of the selected municipalities. Pregnant women were selected via obstetric clinics of the selected municipalities. Doctors, social workers, gynaecologists, and midwifes were permitted to invite their first 100 patients during consultation. A minimum sample size of 130 subjects per target population was calculated. Food consumption data were collected by means of an online, self-administered food frequency questionnaire (FFQ), developed in Lime Survey (https://vitamine.wiv-isp.be) [23]. Since dietary habits rapidly change during infancy and the period of pregnancy and lactation are limited in time, food consumption interviews were conducted with a recall period of one month. Consumption of supplements with vitamins A, D, E, and K, as well as less frequently eaten foods with a high content of fat-soluble vitamins (such as liver or liver products (e.g., pâté)), were examined over the past year. For infants (0–11 months), this was translated into lifetime supplement consumption. To ensure participation of computer or internet illiterate individuals, respondents were offered the possibility to answer the questionnaire with a dietician by phone. Consumption data on infants and toddlers were collected through their proxy, the child’s mother, in most cases.

The questionnaire was adapted to the specific diet of the target populations. The websites of the CHCC-organisation were consulted to derive the food items of a typical infant and toddler menu [24,25]. As such, three different questionnaires were available: a questionnaire for infants, for toddlers, and for adult women. The food list of each questionnaire was composed of food items from the top 90% food groups that contribute to the total intake of vitamin A, D, E, and K in a general adult population. This list was extended with supplements and food brands fortified with vitamin A, D, E, and/or K, identified during a market study [14]. Since fortification (levels) differ in function of the brand of a food item, the food list of the FFQ was detailed up to level of the food brands, in which brand pictures were used to ensure the exact identification of the consumed products. Consumed portion sizes were enquired by means of portion depictions from Globo-diet, as well as pictures of household measures (e.g., glass, spoon …) and food units (e.g., 1 apple, can …). Globo-diet is a standardised program frequently used in European national dietary surveys to assess dietary intake (24 h-recall) [26]. Respondents were asked to encode the kind of fat and/or brand of margarine used for preparation. Fat uptake in the toddler and adult questionnaire was calculated based on the weight yield factors of Bognar et al. [27]. For the preparations of baby meals, parents are advised to add 10–15 g of fat at the end of preparation. Therefore, a portion of 12.5 g of fat was taken into account in the infants questionnaire [28].

### 2.2. Food Composition

The food composition table of the VITADEK study was primarily based on the Belgian food composition table, NUBEL, and the Dutch composition table, NEVO [29,30]. Values for vitamin A, β-carotene, and vitamins D, E, and K were retrieved for each food item included in the FFQ. Missing values were filled with data from four other national food composition tables in the following order: (1) McCance and Widowson’s, the UK food composition table [31]; (2) Ciqual, the French composition table [32]; (3) the Danish food composition table [33]; and (4) the American food composition table [34]. In case of exhaustion of these tables, missing data were filled through ingredient-based or recipe-based calculations, through similarities or as a median value [14]. Missing values for β-carotene and vitamin E in baby-food were analysed in the nutriFOODchem-lab of Ghent University. Breastmilk composition was retrieved from the NEVO-database [29].

In 2015, a market study was conducted to make an up-to-date inventory of vitamins A, D, E, and/or K-fortified foods and supplements available on the Belgian market intended for the target populations under study. Values of the fortified nutrients, labelled on the packages, were collected to complete the food composition table. These values reflect the total content of the fortified nutrient, as they do not differentiate between the naturally occurring amount and what is added during fortification [14]. Composition-data of supplements were obtained from Farmacompendium 2014 (an online database for pharmaceutical products in Belgium), from label information or using online data sources [35].

### 2.3. Fortification and Supplementation in Belgium

Infant and young child nutrition in Belgium is regulated by Royal Decree ((KB) 18/02/1991) and, recently, by European legislation (EU 2013/609 and 2016/127) [19,36]. Regulatory requirements for infant milk and follow-on formula include minimal and maximal doses for vitamins A, D, E, and K. In cereal-based food, intended for infants and young children, a maximum of 3 mg α-TE per 100 kcal is allowed, while addition of other vitamins requires notification. Adding vitamins A and D is not allowed in other baby food, while for vitamin E, the same maximum amounts are set as in cereal-based baby food.

Growing-up milk is not considered a baby food but classified as a voluntarily fortified food, which requires notification (KB 3/3/1992) [36]. In Belgium, vitamin A and D fortification of margarines (fat content (FC) > 80%) and spreadable fats (39% < FC < 41%) (KB 02/10/1980) is mandatory, while fortification of spreadable fats with other fat contents is officially encouraged [20]. Fortification levels vary from 700–900 μg/100 g for vitamin A and 6–7.5 μg/100 g for vitamin D.

The Belgian Superior Health Council (BSHC) recommends vitamin D supplementation of 10 μg/day throughout childhood (0–10 years). However, in practice, recommendations of vitamin D prophylaxis vary according to maternity and which CHCC the child attends. In the Flemish part of Belgium, a supplement of 10 μg/day is recommended (15 μg/day in dark-skinned children), independent of the type of milk nutrition [18]. In the French-speaking region of Belgium, a supplement of 15–20 μg/day is recommended for formula-fed infants and 25–30 μg/day for breastfed infants [37,38].

In Belgium, 1 mg of vitamin K is administered prophylactically (oral or intramuscular) to all newborns, followed by 25 μg /day or 1 mg/week of vitamin K for all breastfed infants until the age of three months [21].

### 2.4. Statistical Analysis

Statistical analyses were performed in SAS 9.3. Nutrient intake was calculated as usual daily consumption by multiplying the frequencies of food consumption by portion size (in g or mL), portion quantities, and nutrient values per 100 g. Population group intake mean and median (P50) values, as well as 5th and 95th percentiles (P5, P95), were computed.

Portion sizes of consumed breast-milk were calculated as the amount of milk corresponding to the energy requirement as a function of age and weight. For infants consuming both formula and breast milk, the amount of breast-milk consumed was estimated as the difference between the recommended amount of breast milk and the volume of formula milk reported [39].

Vitamin A intake was calculated based on conversion factors used by EFSA and expressed in μg retinol equivalents (RE). In this study, however, only retinol and β-carotene were considered [3]. The vitamin K intake values reported in this study represent the total vitamin K intake, i.e., phylloquinones (vitamin K1) plus menaquinones (vitamin K2).

To evaluate the adequacy of the intake of fat-soluble vitamins, intake distributions were evaluated against the dietary reference values set by the EFSA [4,22]. Intake data of vitamin A and retinol were compared to the estimated average requirement (EAR) to evaluate the proportion of the population with inadequate intake (% < EAR) [40].

For vitamins D, E, and K, an Adequate Intake (AI) was used, instead of an EAR, as there is insufficient evidence to determine the distribution of requirements. The evaluation of inadequate intakes with an AI is a qualitative evaluation. When the median intake value of the population is greater than the AI for a certain vitamin, the risk of inadequate intake of this vitamin is considered low. Otherwise, no statement can be made [40].

Excessive intakes were estimated as the proportion of the target population for whom the usual intake exceeds the Upper Limit (UL). For vitamin A, the UL is based on retinol only, due to insufficient scientific basis to set an UL for β-carotene [22].

As a socio-economic parameter, the educational level was evaluated at household level, i.e., the highest educational degree of the parents (infants and toddlers) or the highest degree of the respondent or her partner (pregnant women and lactating women) [14].

## 3. Results

### 3.1. Population Characteristics

Our final study sample consisted of 455 infants, 265 toddlers, 161 pregnant women, and 165 lactating women. Socio-demographic characteristics of the study population are presented in Table 1.

Our study population was gender representative and fairly representative of region (Belgian birth population 2013: Flanders 58%, Walloon region 32%, Brussels capital region 10%) [41]. However, the Brussels capital region was underrepresented in lactating women. Compared to the characteristics of the Belgian population (no degree, primary, or secondary: 41%; higher education of short type: 29.5%; higher education of long type: 29.5%), our study sample showed an underrepresentation of the lowest educational level in favour of the highest one.

An estimated 32% of infants 0–6 months and 16% of infants 7–11 months were breastfed, while 68% and 84%, respectively, received formula or a combination of breastfeeding and formula.

### 3.2. Supplement Consumption

Vitamin D was the most consumed supplement in infants and toddlers (68% and 70%, respectively). Pregnant women and lactating women (67% and 77%, respectively) consumed multivitamins, predominately.

In supplement users 88%, 77%, 61%, and 58% of infants, toddlers, pregnant women, and lactating women, respectively, consumed supplements on medical advice (CHCC, doctor). Other reasons for supplement consumption included (in decreasing order): ‘resistance against diseases,’ ‘being good for health’, and ‘worry about inadequate intake’.

### 3.3. Vitamin Intake and Adequacy

**Figure 1:**
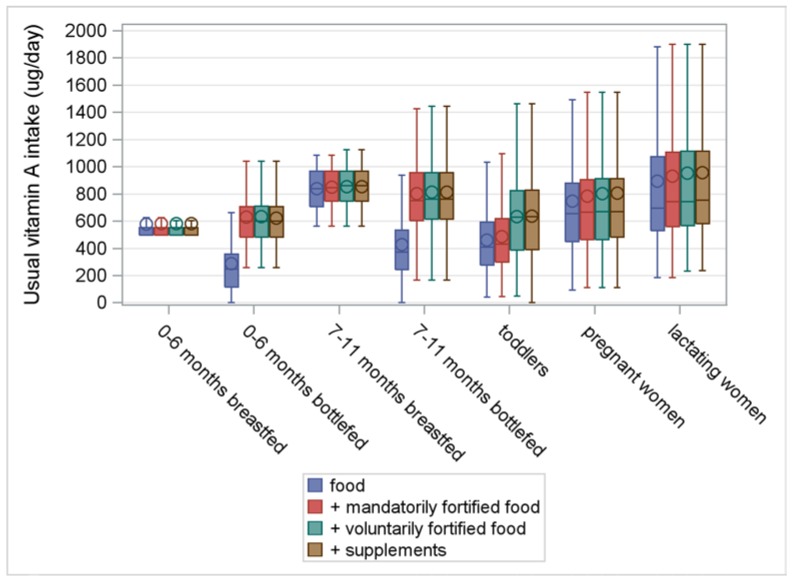
Usual intake of vitamin A (μg/day) from food, fortified food, and supplements in infants, toddlers, pregnant women, and lactating women from a Belgian study on the intake of vitamins A, D, E, and K (VITADEK-study) 2015.

**Figure 2:**
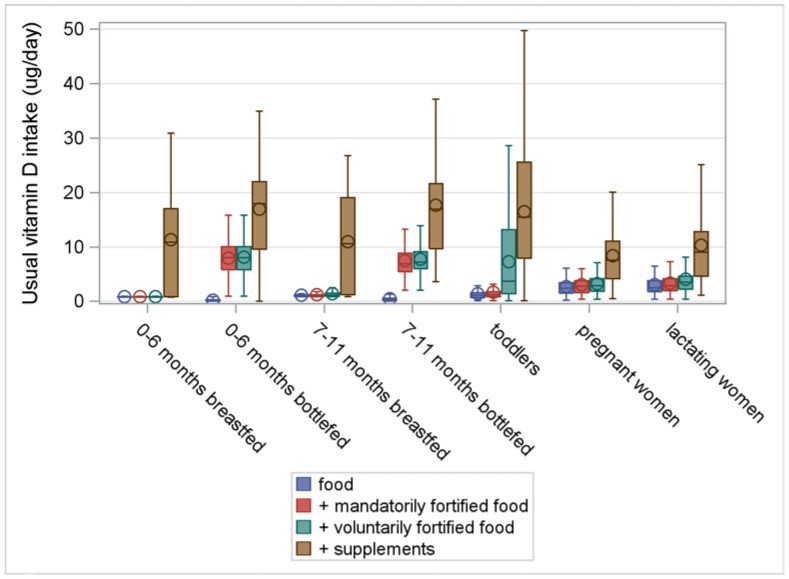
Usual intake of vitamin D (μg/day) from food, fortified food, and supplements in infants, toddlers, pregnant women, and lactating women from a Belgian study on the intake of vitamins A, D, E, and K (VITADEK-study) 2015.

**Figure 3:**
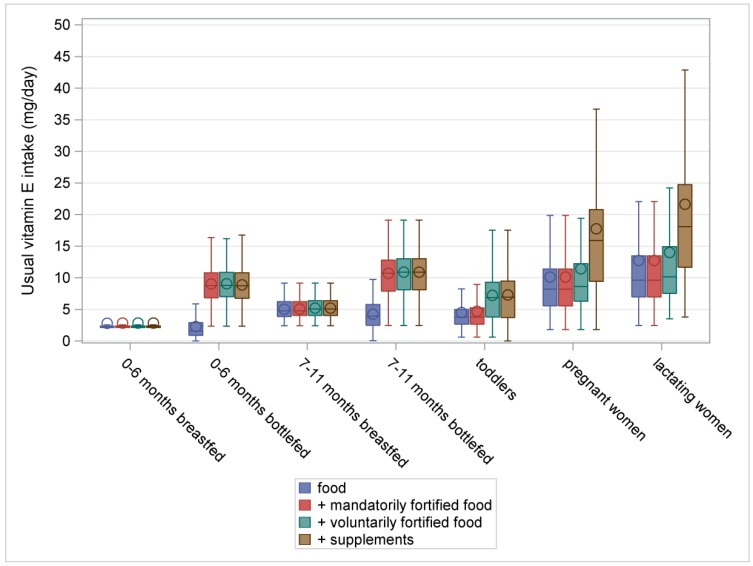
Usual intake of vitamin E (mg/day) from food, fortified food, and supplements in infants, toddlers, pregnant women, and lactating women from a Belgian study on the intake of vitamins A, D, E, and K (VITADEK-study) 2015.

**Figure 4:**
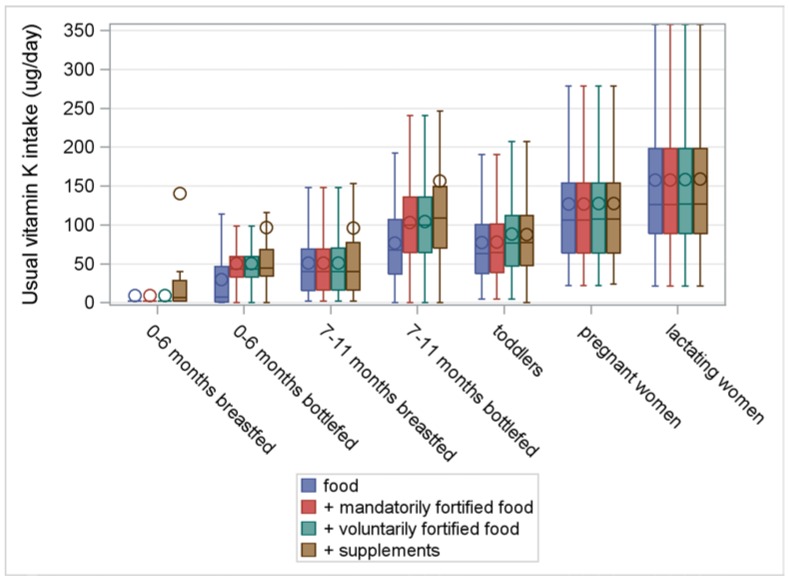
Usual intake of vitamin K (ug/day) from food, fortified food, and supplements in infants, toddlers, pregnant women, and lactating women from a Belgian study on the intake of vitamins A, D, E, and K (VITADEK-study) 2015.

Figure 1, Figure 2, Figure 3 and Figure 4 illustrate the intake distributions and estimates of inadequacy and excess (% < EAR or AI and% > UL) for vitamins A, D, E, and K in Belgian infants, toddlers, pregnant women, and lactating women. The corresponding intake data and percent contributions of each source within the specific subgroup are presented in Supplementary Materials Tables S1–S6, additionally.

#### 3.3.1. Infants

Belgian infants, 0–11 months, met the dietary requirements for all fat-soluble vitamins (Supplementary Materials Tables S1 and S2 and Figure 1, Figure 2, Figure 3 and Figure 4). Breast milk covered the needs for vitamin A in all infants and for vitamin K in infants 7–11 months. However, supplements were indispensable to achieving an adequate vitamin D intake in all infants and for vitamin K in infants 0–6 months. Due to the higher composition of fat-soluble vitamins in formula compared to breast milk, median intakes were higher in formula-fed infants compared to breastfed infants. In the former, mandatorily fortified foods (i.e., infant milk and follow-on milk) delivered adequate amounts of vitamin A, E, and K. However, in high consumers of formula milk (P50 = 720 mL/day; P95 = 1050 mL/day), 1% of infants aged 0–6 months and 6% of infants aged 7–11 months, were at risk for excessive intakes of retinol.

Due to the limited amount of voluntarily fortified foods for this age group (milk-based cereals and cooking fats), their contribution to the total intake of fat-soluble vitamins was insignificant. Supplements made an important contribution to the intakes of vitamin D and K. In breastfed infants, supplements contributed 92% and 87% to the total intake of vitamin D in infants 0–6 months and 7–11 months, respectively. In formula-fed infants, contribution of supplements was 53% and 56% of total vitamin D intake (0–6 months and 7–11 months, respectively). However, inclusion of supplements in the population intake estimates showed that 6% and 17% of breastfed infants and 17% and 26% of formula-fed infants (0–6 months old and 7–11 months old, respectively) were at risk for excessive intake of vitamin D. In breastfed infants, supplements contributed 77% (0–6 months) and 16% (7–11 months), respectively, to the total intake of vitamin K, while in formula-fed infants, contribution of supplements to the total intake of vitamin K was somewhat lower, i.e., 18% (0–6 months) and 10% (7–11 months), respectively.

#### 3.3.2. Toddlers

Mean vitamin A intake from food alone was 479 μg/day and inadequate for 11% of toddlers (Supplementary Materials Table S3 and Figure 1, Figure 2, Figure 3 and Figure 4). In addition to the important contribution of food (69%), mandatorily and voluntarily fortified foods contributed 13% and 18%, respectively, to the total intake of vitamin A. However, when considering all sources, 5% of toddlers remained at risk for inadequate vitamin A intake. Excessive intakes of retinol (% > UL) were related to the consumption of liver and crème pâté (Figure 1, Supplementary Materials Table S6).

The median vitamin D intake from all sources was 16 μg/day, resulting in a low risk of inadequate intake in toddlers. Since mean vitamin D intake from food was very low (1.3 μg/day, or only 9% of the AI), the contribution from mandatorily and voluntarily fortified foods (9% and 28%, respectively) and supplements (55%) was important to achieve adequate vitamin D intakes in toddlers. Voluntarily fortified foods consumed by toddlers included growing-up milk, fortified milk, milk substitutes, multivitamin juices, some dairy desserts, and breakfast cereals.

In toddlers, the risk for inadequate intake of vitamin E from all sources was low. Median intake from food was 3.8 mg/day, or 63% of the AI. Inclusion of mandatorily and voluntarily fortified foods raised median vitamin E intake above the AI (P50 = 7.8 mg/day) with no risk for excessive intakes.

The prevalence of inadequate intakes for vitamin K in toddlers was low. Despite a contribution of 6% from voluntarily fortified foods and 30% from supplements towards total intake, vitamin K requirements in toddlers could be fulfilled with food alone.

#### 3.3.3. Pregnant Women

When considering all sources, 16% of pregnant women had an inadequate intake of vitamin A (<540 μg/day) (Supplementary Materials Table S4 and Figure 1, Figure 2, Figure 3 and Figure 4). The mean intake from food alone was 746 μg/day, or 93% of the total intake of vitamin A. The contribution of mandatorily fortified foods (i.e., margarines and spreading fats) and voluntarily fortified foods was low (5% and 2%, respectively), while contribution of supplements to the total intake of vitamin A was insignificant (Figure 4).

Median vitamin D intake from all sources was 7.6 μg/day, or only 51% of the AI. Due to the limited food sources for vitamin D, median intake from food was only 2.5 μg/day (32% of the total intake). Supplements made the greatest contribution to the total intake of vitamin D (61%), while the contribution of mandatorily (i.e., margarines and spreadable fats) and voluntarily fortified foods was limited (total 7%).

The risk for inadequate intake of vitamin E and K in pregnant women was low. When considering all sources, median vitamin E intake was 15.9 mg/day (AI = 11 mg/day) and median vitamin K intake was 107 μg/day (AI = 70 μg/day). The vitamin intake from foods was sufficient to meet the requirements for vitamin K. For vitamin E, the contribution of supplements was important (36%) to increase the median vitamin E intake to an adequate level.

There was no risk of excessive intakes of vitamins E, D, and K in pregnant women. Higher intakes of retinol resulted from the consumption of crème pâté.

#### 3.3.4. Lactating Women

When considering all sources, 35% of breastfeeding women had an inadequate intake of vitamin A (Supplementary Materials Table S5 and Figure 1, Figure 2, Figure 3 and Figure 4). Food was the major contributor to vitamin A intake (93%) but was insufficient to meet the increased need during lactation. Mandatorily fortified foods (i.e., margarines and spreadable fats) and voluntarily fortified foods contributed only 4% and 2%, respectively, to the total intake of vitamin A).

The median vitamin D intake in lactating women from all sources was 9 μg/day, or only 60% of the AI. The intake from foods was minor (P50 = 2.7 μg/day, or 18% of the AI). Supplements made an important contribution to the total intake of vitamin D (61%), while the contribution of mandatorily and voluntarily fortified foods was limited to 3% and 7%, respectively, of the total intake.

Lactating women had a low risk for inadequate intake of vitamin E from all sources. Median vitamin E intake from food was below the vitamin E requirements (P50 = 10 mg/day). An important contribution was made by voluntarily fortified foods and supplements (6% and 38%, respectively), bringing the median intake to 18 mg/day.

Median vitamin K intake from all sources was 126 μg/day, which was far above the AI of 70 μg/day. The need for vitamin K was completely fulfilled by the intake from food. Since the consumed fortified foods and multivitamins did not contain vitamin K, their contribution to the total intake of vitamin K was insignificant.

## 4. Discussion

For the first time, the VITADEK-study presents the intake of fat-soluble vitamins in four vulnerable groups of the Belgian population—infants, toddlers, pregnant women, and lactating women. The study is unique, as it evaluates the contribution from all sources on population intake distributions through a quantitative assessment of current Belgian fortification and supplementation practices.

The study revealed inadequate vitamin A intakes in 5% of toddlers, 16% of pregnant women, and 35% of lactating women. Median vitamin D intake in pregnant women and lactating women was only half of the AI, despite good supplement compliance. The risk for inadequate vitamin D intake was low in infants and toddlers. However, non-users of vitamin D prophylaxis in infants and toddlers were at risk for vitamin D inadequacy, while supplement consumption posed a risk for excessive intake in 6%–26% of infants. In toddlers alone, voluntarily fortified foods made an important contribution to the intake of fat-soluble vitamins. In all studied populations, the risk for inadequate and excessive intakes of vitamin E and K was considered low. Therefore, the discussion section mainly focuses on vitamin A and D intake.

### 4.1. Vitamin A

#### 4.1.1. Infants and Toddlers

During infancy, the dominantly milk-based diet (breastmilk and/or formula) covers the needs for vitamin A [3]. However, it should be stipulated that for breastmilk composition, no biological samples were collected, but a mean vitamin A content was used [3]. In the first months of life, the proportion of preformed retinol is high, but decreases in favor of β-carotene (with lower bioeffectiveness, i.e., 1RE = 1/6 β-carotene) due to the introduction of vegetables and fruits [42]. This explains the lower median vitamin A intake in our toddler sample compared to infants aged 7–11 months. Our results in toddlers were in line with the European review report of Mensink et al. [43]. Inadequate intake of vitamin A in toddlers was also reported in English, Dutch, and Flemish pre-schoolers [43]. Unbalanced consumption of fruits, vegetables, and fish are most likely at the base of these vitamin A inadequacies [44,45,46].

Consistent with our results, excessive vitamin A intakes in European infants were frequently reported in high consumers of formula [42]. Reported mean/median vitamin A intakes in formula-fed infants ranged from 510 to 980 μg/RE/day in infants 0–6 months and 530 to 1090 μg RE/day in infants 7–12 months [47]. Although clinical symptoms appear at much higher doses of 450 μg RE/kg bodyweight/day, a new EU regulation, which aims to eliminate the risk of excessive intakes by reducing maximum levels, will be enforced in 2020 [19,42]. Milk consumption in high consumers of formula was high compared to the recommended amounts for this age. However, as data were self-reported, we cannot exclude overestimation of the consumed amount of milk and related intakes of vitamin A in formula-fed infants. The excessive vitamin A intake in toddlers, resulting from the consumption of liver and crème pâté, was also observed in a Dutch study [44].

#### 4.1.2. Pregnant Women and Lactating Women

Inadequate vitamin A intake in pregnant women was consistent with our report of the general Belgian population. In Belgian women, 15% had an inadequate intake of vitamin A, which may be related to the unbalanced consumption of vegetables and dairy products [16]. The higher proportion of inadequate intake in lactating women is, therefore, not surprising, considering the additional requirement of 530 μg/day during lactation [3].

Vitamin A deficiency is rare in industrialised countries. Evidence demonstrates that both vitamin A supply to the foetus and retinol concentrations in breastmilk are carefully regulated to support physiological development and are relatively insensitive to maternal status (except in poorly nourished mothers). However, in case of prolonged breastfeeding (6 months), retinol concentration in breastmilk from mothers with low vitamin A intake might be inadequate [48]. Targeted screening of vitamin A status, using stable isotopes, is needed to substantiate the public health impact of low dietary intake in Belgium [3,49]. Meanwhile, it is beneficial for both mother and breast-fed infant to improve their vitamin A status. Promoting a well-balanced and vitamin-A riche diet or a well-considered vitamin A fortification of foods are the safest ways to obtain adequate vitamin A intake. Unlike Belgium, where retinol intake from supplements is strongly discouraged due to the teratogenic effect, supplements significantly reduced inadequate vitamin A intake in Spanish pregnant women [50].

### 4.2. Vitamin D

Due to the limited amounts of vitamin D food sources, intake from food is low. The richest food sources, such as oil-rich fish, offal, and egg yolk, are neither common in infants’ or toddlers’ menus, nor preferred dishes in Belgian dietary habits.

#### 4.2.1. Infants and Toddlers

In both breastfed and formula-fed infants, vitamin D intake from normal and fortified foods was inadequate. Despite breast milk being the recommended food source for infants, its average vitamin D content is very low (0.25–2.0 μg/L in un-supplemented and supplemented women, respectively), resulting in inadequate intake levels [2]. In Belgium, infant milk and follow-on milk are fortified with vitamin D, resulting in an average dose of 10 μg/L. Considering the consumed amount of milk, vitamin D intake from formula does not cover the recommended 10 μg/day, as was also observed in other studies. The new regulation on infant formula and follow-on formulas, applicable in 2020, will increase the minimum level of vitamin D from 1 to 2 μg/100 kcal but will keep the maximum level at 3 μg/100 kcal. This will result in an increase of vitamin D intake in our study population, as current median vitamin D content of the consumed formula was 1.8 μg/100 kcal [18].

The median vitamin D intake from foods and fortified foods in toddlers was inadequate and lower than in formula-fed infants. Beyond 12 months of age, other types of milk often replace formula (fortified) milk. In our study, 45% of toddlers consumed growing-up milk, enriched milk, or milk substitutes fortified with vitamin D levels similar to formula (1.2 μg/100 mL to 3.1 μg/100 mL), while others consumed whole milk or semi-skimmed milk, which does not contain vitamin D [29]. A study in the UK indicated that fortified milks could play a significant role in improving the diet of young children [12].

In the present sample, vitamin D supplements helped achieve adequate intake in both infants and toddlers. However, 16% of the breastfed infants, 31% of the formula-fed infants, and 30% of toddlers never took vitamin D supplements. In breastfed infants, especially, this is a major concern due to the very low intake through breastmilk [18]. Prevailing studies on infants and toddlers report high prevalence of vitamin D insufficiency (S-25(OH)D < 30 nmol/L), mainly in non-users of vitamin D prophylaxis [18,51]. Inadequate vitamin D intake in these age-groups may lead to diminished bone mineralisation and, in extremis, to rickets, thus stressing the need for vitamin D supplementation [2,8,18].

Vitamin D synthesis in the skin, under influence of UVB-irradiation, is the major source of vitamin D. Therefore, judicious sun-exposure should be encouraged to prevent vitamin D deficiency [8]. However, beside environmental factors, personal factors such as skin pigmentation, use of sunscreen, and sun exposure behaviour may impede vitamin D synthesis. Young children, for whom direct sun-exposure is strongly discouraged, are therefore more vulnerable to vitamin D deficiency. The beneficial effect of vitamin D prophylaxis to maintain year-round S-25(OH)D levels stresses the need for vitamin D supplementation [18]. Analysis of socio-demographic and lifestyle characteristics is required to identify non-users of vitamin D prophylaxis to allow a target screening and treatment of non-users. Additionally, fortification should be investigated as an alternative strategy to prevent vitamin D deficiency, especially since reliance on vitamin D prophylaxis decreases with age [16]. Lastly, country-wide surveys on vitamin D status are required for infants and toddlers to substantiate the magnitude of inadequate vitamin D intake.

Besides inadequate vitamin D intakes, a substantial proportion of Belgian infants were at risk for excessive intakes of vitamin D. The UL for vitamin D is based on the non-observed adverse health effect level (NOAL) for adolescents. However, there is no consensus in setting this NOAL. EFSA proposes an UL for vitamin D of 50 μg/day by taking into account their smaller body size. However, since evidence is lacking to prove the absence of toxicity at 50 μg/day, the Norwegian committee maintains an UL of 25 μg/day [2,52]. This latter recommendation is followed by the BSHC [52]. The lack of consensus in the Belgian supplementation guidelines reflects this uncertainty and might explain intakes exceeding the UL. Adverse health effects are not expected in our study population, since large safety factors were used in setting this NOAL [22,53]. However, there is a need for consensus on vitamin D supplementation, in awaiting a uniform setting of the UL.

#### 4.2.2. Pregnant Women and Lactating Women

Low vitamin D intakes in our study population of pregnant women and lactating women are explained by the limited food sources for vitamin D and the limited consumption of foods fortified with vitamin D (the latter results from the fact that the Belgian market of foods fortified with vitamin D is limited and mainly targets children and adolescents [16,54]). The consumption of vitamin D-containing supplements did not contribute to an adequate intake of vitamin D, despite high supplement compliance. This might be related to the vitamin D content of the consumed multivitamins (P50 = 400 IU or 10 μg), which is lower than the AI of 600 IU or 15 μg/day recommended by EFSA [2]. These findings confirm the previously reported results on Belgian pregnant women and have not changed since [17].

Inadequate vitamin D intakes do not predispose individuals to vitamin D deficiency. However, the high prevalence of vitamin D insufficiency (S-25(OH)D < 30 nmol/L) in Belgian pregnant women and, more generally, in European pregnant women and lactating women, is a corollary of insufficient sun-exposure. This results from our increased sedentary lifestyle with fewer outdoor activities. Therefore, judicious sun-exposure should be better promoted. Additionally, since our results showed a good supplement adherence, a well-considered increase in the vitamin D content of multivitamins might be a possible strategy to correct this deficiency. A first and safe approach might be to increase the vitamin D content of these multivitamins for pregnant women up to a level equal to the AI. Obviously, scenario-studies are needed to investigate the possible effect of fortification or increased supplement contents.

### 4.3. Vitamin E

Our study reported adequate vitamin E intake in each of the subgroups. We compared our data to that of EFSA, using the EFSA Comprehensive European Food Consumption Database and the EFSA Food Composition Database. In infants aged 1–11 months, average α-TE intakes ranged between 3.2 and 5.9 mg/day. In children aged 1 to 3 years, α-TE intakes ranged between 4.4 and 7.3 mg/day. When recalculating our data for all infants (breastfed and formula-fed) aged 0–11 months, mean vitamin E intake from all sources was 8.19 mg/day, somewhat higher than the review data. Our data for toddlers was consistent with the EFSA’s findings [1].

In our study, vitamin E intake in pregnant women was higher than the mean intake reported in Latvian pregnant women. However, these data do not include intake from supplements. Mean α-TE intakes in European lactating women varied between 14.9 mg/day and 16.95 mg/day. Our results were somewhat higher than in the EFSA review study (39%–52% were supplement users), probably due to a higher supplement use in our study population of lactating women.

### 4.4. Vitamin K

Mean vitamin K estimates of the EFSA database without supplements ranged between 23 and 61 μg/day in infants (<1 year) and between 36 and 53 μg/day in children aged 1 to <3 years. Mean vitamin K intake recalculated for our entire infant population, 0–11 months, was 61 g/day and thus in line with the EFSA estimates.

The vitamin K intake in our toddler study-population was substantially higher than the EFSA estimates [4]. This might be explained by a higher contribution of vegetables containing high doses of vitamin K. The mean portion size of consumed vegetables was 90 g (P50 = 81 g, P95 = 196 g). These values are high compared to a study in Flemish pre-schoolers 2.5–4 years and the recommended portion sizes for this age, between 100 and 150 g [45]. Response bias cannot be excluded, since respondents might have had a problem recalling the type, frequency, and portion of vegetables consumed in the past months. However, since median vitamin K intake is more than fivefold the AI of 12 μg/day, vitamin K intake in toddlers was evaluated as adequate. Our results in pregnant women and lactating women were in line with EFSA data of women of reproductive age (P50 ranges between 72 and 196 μg/day). In the scientific opinion of dietary reference values for vitamin K, EFSA concluded that the AI for women of reproductive age also applies to pregnant women and lactating women. Available data could not justify an additional need for vitamin K during pregnancy, while the limited excretion of vitamin K (mainly phylloquinone) in breast milk could also be covered by the AI for women aged 18–39 [4].

Finally, as reported by EFSA, it should be noted that due to methodological limitations, considerable uncertainties prevail in the available dietary intake estimates for vitamin K [4].

### 4.5. Strengths and Limitations

A strength of our study is the simultaneous evaluation of the intake of fat-soluble vitamins in four vulnerable groups, as each has different requirements and risk exposure. Next, the study evaluates the contribution of all sources, allowing a more precise estimate of intake distributions. Fortified food and supplements can be an important source of variation in the intake estimates, especially for vitamin D [55].

We previously reported that our study population had a greater proportion of participants with higher educational level than expected. An unbalanced food consumption or non-adherence to supplement advice is likely to be associated with educational degree [11,56]. Therefore, we cannot exclude an overestimation of the population intake estimates.

Another limitation of the study was the lack of validation of the FFQ, due to budgetary constraints. By using the BFCS as a basis for the construction of the questionnaires (selection of food items and brands, use of the same portion depictions), we tried to overcome some well-known limitations of the FFQ [57,58]. As a result, our data in infants and toddlers connect with the youngest age group of the BFCS, while our results in pregnant women and lactating women were in line with the data from women of reproductive age from the BFCS. A difficult task remained for the respondents to recall the consumed portion frequency and size over the past month. Overestimations of consumed amounts are observed when respondents tend to answer what is socially desirable [58,59]. In our study, this was reflected in the over-reporting of vegetable consumption and was taken into account when discussing these findings.

Finally, the data collected with a FFQ should reflect the respondents’ usual diet. A recall period of one month for food was deemed sufficient, due to the frequency of consumption of vitamins A, D, E, and K-fortified foods (breakfast cereals, milk, dairy products, and fruit juices). For supplements, the recall period of one year covers the seasonal variability in supplement intake. However, for consumed foods, we were not able to consider the seasonal variability.

## 5. Conclusions

We present the first evaluation of the risk for inadequate and excessive intakes of vitamins A, D, E, and K in Belgian infants, toddlers, pregnant women, and lactating women. Vitamin E and K intake from all sources was adequate in all subgroups under study. For vitamin A and D, both inadequate and excessive intakes were reported. Current fortification and supplementation practices, with respect to vitamins A and D, need to be revised. To obtain adequate vitamin D intakes in Belgian infants, toddlers, pregnant women, and lactating women, we recommend a targeted screening to detect and treat non-users of vitamin D prophylaxis. Scenario studies are needed to evaluate the effect of possible vitamin A and D fortification strategies as an alternative or as an additional measure of supplementation programs. Finally, there is a need for setting uniform national recommendations for vitamin D prophylaxis in Belgian infants and toddlers.

## Figures and Tables

**Table 1:** Characteristics of the study population in a Belgian study of the intake of vitamins A, D, E, and K (VITADEK-study) 2015.

	Gender/Duration Pregnancy/Age Infant (%)		Age (%)		Region (%)		Nationality Respondent/Proxy (%)		Educational Level (Highest Degree of Parents/Respondent or Partner) (%)	
Infants	Total	455								
	Boys ^(1)^	227 (50)	0–3 months	153 (34)	Flanders	265 (58)	Belgian	387 (85)	No degree/secondary	98 (22)
	Girls ^(1)^	228 (50)	4–6 months	156 (34)	Brussels capital region	43 (9)	EU resident (non-Belgian)	30 (7)	Bachelor	144 (32)
			7–11 months	146 (32)	Wallonia	147 (32)	Non-EU resident	38 (8)	Master	213 (47)
Toddlers	Total	263								
	Boys ^(1)^	135 (51)	12–23 months	176 (67)	Flanders	124 (60)	Belgian	228 (87)	No degree/secondary	64 (24)
	Girls ^(1)^	128 (49)	24–35 months	87 (33)	Brussels	20 (10)	EU resident (non-Belgian)	12 (4)	Bachelor	81 (31)
					Wallonia	61 (30)	Non-EU resident	23(9)	Master	118 (45)
Pregnant women	Total	145								
	1st trimester ^(2)^	17 (12)	19–30 years	78 (54)	Flanders	109 (75)	Belgian	130 (90)	No degree/secondary	25 (17)
	2nd trimester ^(2)^	60 (41)	31–40 years	64 (44)	Brussels	7 (5)	EU resident (non-Belgian)	9 (6)	Bachelor	46 (32)
	3rd trimester ^(2)^	68 (47)	>41 years	3 (2)	Wallonia	29 (20)	Non-EU resident	6 (4)	Master	74 (51)
Lactating women	Total	147								
	infant: 0–3 months ^(3)^	86 (59)	19–30 years	76 (52)	Flanders	85 (58)	Belgian	124 (84)	No degree/secondary	26 (18)
	Infant: 4–6 months ^(3)^	33 (22)	31–40 years	70 (48)	Brussels	13 (9)	EU resident (non-Belgian)	10 (7)	Bachelor	46 (31)
	Infant: 7–12 months ^(3)^	28 (19)	>41 years	1 (1)	Wallonia	49 (33)	Non-EU resident	13 (9)	Master	75 (51)

^(1)^, Gender; ^(2)^, Duration pregnancy; ^(3)^, Age infant.

## Supplementary Materials

The following are available online at http://www.mdpi.com/2072-6643/10/2/223/s1, Table S1: Usual intake of vitamin A, D, E and K (μg/day) from food, fortified food and supplements in infants (0–6 months), Belgian study on the intake of vitamins A, D, E and K (VITADEK-study) 2015; Table S2: Usual intake of vitamin A, D, E and K (μg/day) from food, fortified food and supplements in infants (7–11 months), Belgian study on the intake of vitamins A, D, E and K (VITADEK-study) 2015; Table S3: Usual intake of vitamin A, D, E and K (μg/day) from food, fortified food and supplements in toddlers (12–35 months), Belgian study on the intake of vitamins A, D, E and K (VITADEK-study) 2015; Table S4: Usual intake of vitamin A, D, E and K (μg/day) from food, fortified food and supplements in pregnant women, Belgian study on the intake of vitamins A, D, E and K (VITADEK-study) 2015; Table S5: Usual intake of vitamin A, D, E and K (μg/day) from food, fortified food and supplements in breastfeeding women, Belgian study on the intake of vitamins A, D, E and K (VITADEK-study) 2015; Table S6: Usual intake of retinol (μg/day) from food, fortified food and supplements in infants, toddlers, pregnant and lactating women, Belgian study on the intake of vitamins A, D, E and K (VITADEK-study) 2015.
