# Supplementary material for: Do Current Fortification and Supplementation Programs Assure Adequate Intake of Fat-Soluble Vitamins in Belgian Infants, Toddlers, Pregnant Women, and Lactating Women?
Source: Nutrients. 2018 Feb 16;10(2):223. doi: 10.3390/nu10020223 (PMC5852799; doi:10.3390/nu10020223)
Supplement: Supplementary file 1 [file nutrients-10-00223-s001.pdf]

Table S1| Usual intake of **vitamin A, D, E and K** (µg/day) from food, fortified food and supplements in **infants (0-6 months)**, Belgian study on the intake of vitamins A, D, E and K (VITADEK-study) 2015.

	Source	EAR/AI[1,2]	UL[3]	Usual intake	% contri bution	5	50	95	% < EAR/ Risk of inade quacy	% > UL	Usual intake	% contri bution	5	50	95	% < EAR/ Risk of inade quacy	% > UL
				**Breastfed infants**							**Formula-fed infants**						
VITAMIN A	Food	180	800	580	99.3	497	551	905	0.00	0.00	283	45.7	10	248	725	36	0.00
	+ mandatorily fortified food	180	800	583	0.5	497	551	905	0.00	0.00	623	53.5	398	600	1005	0.00	0.97
	+ voluntarily fortified foods	180	800	584	0.2	497	551	905	0.00	0.00	627	0.8	398	601	1012	0.00	1.45
	+ supplements	180	800	584	0	497	551	908	0.00	0.00	627	0	398	601	1012	0.00	1.45
VITAMIN D	Food	10	25	0.84	7.4	0.78	0.87	1.01	ns	0.00	0.22	0.6	0.00	0.10	0.91	ns	0.00
	+ mandatorily fortified food	10	25	0.87	0.3	0.78	0.87	1.24	ns	0.00	7.93	45.6	3.02	8.01	13.50	ns	0.00
	+ voluntarily fortified foods	10	25	0.89	0.2	0.78	0.87	1.24	ns	0.00	8.03	0.6	3.02	8.01	13.65	ns	0.00
	+ supplements	10	25	11.36	92.2	0.78	10.9	25.49	low	6.06	16.82	53.2	4.19	17.96	31.48	low	16.67
VITAMIN E	Food	4	100	2.82	99.3	2.13	2.36	6.47	ns	0.00	2.27	25.3	0.06	1.57	7.76	ns	0.00
	+ mandatorily fortified food	4	100	2.82	0.7	2.13	2.36	6.47	ns	0.00	8.93	74.2	3.95	8.76	14.71	low	0.00
	+ voluntarily fortified foods	4	100	2.84	0	2.13	2.36	6.58	ns	0.00	8.98	0.6	3.98	8.77	14.71	low	0.00
	+ supplements	4	100	2.84	99.3	2.13	2.36	6.58	ns	0.00	8.98	0	3.98	8.77	14.71	low	0.00
VITAMIN K	Food	5		9.28	23.3	2.13	2.36	40.29	ns		29.5	47.9	0.00	7.38	124.8	low	
	+ mandatorily fortified food	5		9.28	0.1	2.13	2.36	40.29	ns		50	33.3	1.32	43.0	127.6	low	
	+ voluntarily fortified foods	5		9.30	0.1	2.13	2.36	40.29	ns		50.3	0.5	1.32	43.0	127.6	low	
	+ supplements	5		39.89	76.7	2.13	6.53	282.1	low		61.6	18.3	2.92	44.8	167.9	low	

(1) For children below 6 months born at full term, there are no reference value based on real needs but adequate intakes are based on observed intakes of exclusively breastfed children. When no value was available (vitamins A and E) the same reference values as for infants 7-11 months were used.

(2) The AI for vitamin K is derived from an AI of 1 µg per kg body weight and an average weight of 5 kg for infants aged 0 to < 6 months (60).

(3) For infants 0-6 months no UL is set for vitamin K.

EAR: Estimated average requirement; AI: Adequate intake; UL: Upper intake level

Table S2| Usual intake of **vitamin A, D, E and K** (µg/day) from food, fortified food and supplements in **infants (7-11 months)**, Belgian study on the intake of vitamins A, D, E and K (VITADEK-study) 2015.

	Source	EAR/AI[1]	UL[1]	Usual intake	% contribution	5	50	95	% < EAR/ Risk of inadequacy	% > UL	Usual intake	% contribution	5	50	95	% < EAR/ Risk of inadequacy	% > UL
				**Breastfed infants**							**Formula-fed infants**						
VITAMIN A	Food	190	800	838	98.1	572	841	1083	0.00	0.00	429	52.6	122	372	935	12.5	0.00
	+ mandatorily fortified food	190	800	850	1.4	601	845	1083	0.00	0.00	801	45.6	433	751	1301	0.83	5.79
	+ voluntarily fortified foods	190	800	854	0.5	601	861	1084	0.00	4.17	815	1.7	447	760	1378	0.83	5.79
	+ supplements	190	800	854	0	601	861	1084	0.00	4.17	815	0	447	760	1378	0.83	5.79
VITAMIN D	Food	10	25	1.10	10.0	0.88	1.03	1.44	ns	0.00	0.51	2.9	0.00	0.28	2.06	ns	0.00
	+ mandatorily fortified food	10	25	1.23	1.2	0.88	1.03	2.14	ns	0.00	7.46	39.3	4.00	6.95	12.08	ns	0.83
	+ voluntarily fortified foods	10	25	1.38	1.4	0.88	1.12	2.51	ns	0.00	7.74	1.6	4.37	7.23	12.68	ns	0.83
	+ supplements	10	25	10.96	87.4	0.88	10.63	26.51	ns/low	16.67	17.70	56.3	5.67	16.92	34.65	low	26.45
VITAMIN E	Food	5	100	5.03	96.7	2.60	4.78	7.82	ns	0.00	4.26	39.1	1.56	3.94	8.70	ns	0.00
	+ mandatorily fortified food	5	100	5.08	1	2.60	4.78	7.82	ns	0.00	10.70	21.3	5.56	10.71	17.04	low	0.00
	+ voluntarily fortified foods	5	100	5.20	3.3	2.61	5.05	7.82	low	0.00	10.89	60.9	6.12	10.88	17.94	low	0.00
	+ supplements	5	100	5.20	0	2.61	5.05	7.82	low	0.00	10.89	0	6.12	10.88	17.94	low	0.00
VITAMIN K	Food	8.5		50.9	83.9	6.4	40.3	148.0	ns		76.57	66	14.6	67.7	168.8	low	
	+ mandatorily fortified food	8.5		51.2	0.5	6.4	40.3	148.0	ns		103.9	23.5	28.0	99.9	199.9	low	
	+ voluntarily fortified foods	8.5		51.2	0	6.4	40.3	148.0	low		104.2	0.3	29.5	99.9	203.3	low	
	+ supplements	8.5		60.7	15.7	6.4	40.3	153.0	low		116.1	10.2	29.5	109.2	203.5	low	

(1) The AI for vitamin K is derived from an AI of 1 µg per kg body weight and an average weight of 8.5 kg for infants aged 7 to 11 months (60).

(2) For infants 7-11 months nu UL is set for vitamin K.

EAR: Estimated average requirement; AI: Adequate intake; UL: Upper intake level

Table S3| Usual intake of **vitamin A, D, E and K** (µg/day) from food, fortified food and supplements in **toddlers (12-35 months)**, Belgian study on the intake of vitamins A, D, E and K (VITADEK-study) 2015.

		AI[1]	UL	Usual intake	% contribution	5	50	95	Risk of inadequacy	% > UL
VITAMIN A	Food	205	800	479	69.2	163	404	1053	11.15	0.8
	+ mandatorily fortified food	205	800	567	12.7	193	507	1129	7.28	2.3
	+ voluntarily fortified foods	205	800	691	17.9	211	666	1265	4.98	3.1
	+ supplements	205	800	692	0.1	204	667	1265	4.98	3.1
VITAMIN D	Food	15	50	1.31	8.1	0.23	1.00	3.41	ns	0.00
	+ mandatorily fortified food	15	50	2.71	8.6	0.47	1.44	8.09	ns	0.00
	+ voluntarily fortified foods	15	50	7.25	28	0.65	5.52	20.04	ns	0.00
	+ supplements	15	50	16.21	55.3	1.36	15.50	32.89	low	0.00
VITAMIN E	Food	6	60	4.70	57.5	1.76	3.80	10.25	ns	0.00
	+ mandatorily fortified food	6	60	6.01	16	1.79	4.40	14.63	ns	0.00
	+ voluntarily fortified foods	6	60	8.13	25.9	2.06	7.78	15.80	low	0.00
	+ supplements	6	60	8.17	0.5	2.05	7.84	15.81	low	0.00
VITAMIN K	Food	12	200	77.99	59.3	21.54	65.62	182.42	low	3.83
	+ mandatorily fortified food	12	200	83.39	4.1	22.49	72.88	186.62	low	3.83
	+ voluntarily fortified foods	12	200	91.42	6.1	25.56	82.28	186.63	low	4.21
	+ supplements	12	200	131.48	30.5	25.10	84.61	207.17	low	4.90

[1] The AI for vitamin K is derived from an AI of 1 µg per kg body weight and an average weight of 12 kg for infants aged 12-35 months (60).

EAR: Estimated average requirement. AI: Adequate intake; UL: Upper intake level

Table S4| Usual intake of **vitamin A, D, E and K** (µg/day) from food, fortified food and supplements in **pregnant women**, Belgian study on the intake of vitamins A, D, E and K (VITADEK-study) 2015.

		EAR/AI[1]	UL	Usual intake	% contribution	5	50	95	% < EAR/Risk of inadequacy	% > UL
VITAMIN A	Food	540	3000	746	92.7	214	656	1710	18	0.34
	+ mandatorily fortified food	540	3000	784	4.7	267	665	1728	17	0.34
	+ voluntarily fortified foods	540	3000	803	2.4	267	669	1821	16	0.34
	+ supplements	540	3000	805	0.2	267	669	1821	16	0.34
VITAMIN D	Food	15	100	2.72	32.4	0.63	2.46	5.82	ns	0
	+ mandatorily fortified food	15	100	3.03	3.7	0.64	2.81	5.95	ns	0
	+ voluntarily fortified foods	15	100	3.29	3.1	0.89	2.86	6.99	ns	0
	+ supplements	15	100	8.40	60.8	1.48	7.58	18.75	ns	0
VITAMIN E	Food	11	300	10.12	57.1	3.00	8.20	16.97	ns	0
	+ voluntarily fortified food	11	300	11.40	7.2	3.34	8.56	21.89	ns	0
	+ supplements	11	300	17.73	35.7	6.41	15.90	34.36	low	0
VITAMIN K	Food	70	1000	127	99.2	36	107	308	low	0
	+ voluntarily fortified foods	70	1000	127	0	36	107	308	low	0
	+ supplements	70	1000	128	0.8	36	107	308	low	0

EAR: Estimated average requirement; AI: Adequate intake; UL: Upper intake level

Table S5| Usual intake of **vitamin A, D, E and K** (µg/day) from food, fortified food and supplements in **breastfeeding women**, Belgian study on the intake of vitamins A, D, E and K (VITADEK-study) 2015.

		EAR/AI[1]	UL	Usual intake	% contribution	5	50	95	% < EAR/Risk of inadequacy	% > UL
VITAMIN A	Food	1020	3000	894	93.4	288	696	2103	36	0.00
	+ mandatorily fortified food	1020	3000	932	4	334	745	2169	35	0.00
	+ voluntarily fortified foods	1020	3000	954	2.3	363	745	2279	35	0.00
	+ supplements	1020	3000	957	0.3	363	755	2279	35	0.00
VITAMIN D	Food	15	100	3.04	29.6	1.12	2.50	7.16	ns	0.00
	+ mandatorily fortified food	15	100	3.36	3.1	1.13	2.88	7.95	ns	0.00
	+ voluntarily fortified foods	15	100	4.05	6.7	1.25	3.49	9.04	ns	0.00
	+ supplements	15	100	10.28	60.6	2.04	9.07	22.68	ns	0.00
VITAMIN E	Food	11	300	11.31	56	4.50	9.64	30.98	ns	0.00
	+ voluntarily fortified food	11	300	12.56	6.2	5.08	10.06	32.38	ns	0.00
	+ supplements	11	300	20.2	37.8	7.67	18.12	44.36	low	0.00
VITAMIN K	Food	70	1000	155	99.4	46	126	358	low	0.31
	+ voluntarily fortified foods	70	1000	156	0.6	51	127	358	low	0.31
	+ supplements	70	1000	156	0	51	127	358	low	0.31

EAR: Estimated average requirement; AI: Adequate intake; UL: Upper intake level

Table S6| Usual intake of **retinol** (µg/day) from food, fortified food and supplements in **infants, toddlers, pregnant and lactating women**, Belgian study on the intake of vitamins A, D, E and K (VITADEK-study) 2015.

		Usual intake	5	50	95	UL	% > UL	Usual intake	5	50	95	UL	% > UL
Infants 0-6 months				Breastfed						Formula-fed			
	Food	528	497	551	578	800	0.00	85	0	7	421	800	0.00
	+ mandatorily fortified food	531	497	551	605	800	0.00	500	288	486	742	800	1.46
	+ voluntarily fortified foods	532	497	551	606	800	0.00	504	295	490	742	800	1.94
	+ supplements	532	497	551	606	800	0.00	504	295	490	738	800	1.94
Infants 7-11 months	Food	580	562	566	642	800	0.00	62.0	0.13	15.5	383	800	0.00
	+ mandatorily fortified food	590	562	569	667	800	0.00	435	233	377	821	800	5.79
	+ voluntarily fortified foods	595	562	578	667	800	4.17	448	255	390	826	800	5.79
	+ supplements	595	562	578	667	800	4.17	448	255	390	826	800	5.79
Toddlers	Food	161	5.37	111	581	800	0.8						
	+ mandatorily fortified food	248	36.6	196	651	800	2.3						
	+ voluntarily fortified foods	368	49.9	367	746	800	2.7						
	+ supplements	370	43.4	373	746	800	3.1						
Pregnant women	Food	294	58	228	666	3000	0.34						
	+ mandatorily fortified food	329	68	248	705	3000	0.34						
	+ voluntarily fortified foods	329	68	252	705	3000	0.34						
	+ supplements	332	68	252	705	3000	0.34						
Lactating women	Food	298	76	251	638	3000	0.00						
	+ mandatorily fortified food	332	101	298	668	3000	0.00						
	+ voluntarily fortified foods	338	101	299	668	3000	0.00						
	+ supplements	342	112	303	668	3000	0.00						

UL: Upper intake level
